# Fracture Load Predictions in Additively Manufactured ABS U-Notched Specimens Using Average Strain Energy Density Criteria

**DOI:** 10.3390/ma15072372

**Published:** 2022-03-23

**Authors:** Marcos Sánchez, Sergio Cicero, Sergio Arrieta, Victor Martínez

**Affiliations:** LADICIM (Laboratory of Materials Science and Engineering), Universidad de Cantabria, E.T.S. de Ingenieros de Caminos, Canales y Puertos, Av/Los Castros 44, 39005 Santander, Spain; sergio.arrieta@unican.es (S.A.); victor.martinezmata@unican.es (V.M.)

**Keywords:** additive manufacturing, ABS, fracture, notch, average strain energy density, equivalent material concept, fictitious material concept

## Abstract

This paper provides a methodology for the prediction of fracture loads in additively manufactured ABS material containing U-notches. The approach is based on the Average Strain Energy Density (ASED) criterion, which assumes that the material being analysed develops fully linear-elastic behaviour. Thus, in those cases where the material has a certain (non-negligible) amount of non-linear behaviour, the ASED criterion needs to be corrected. In this sense, in this paper, the ASED criterion is also combined with the Equivalent Material Concept (EMC) and the Fictitious Material Concept (FMC), both being corrections in which the non-linear real material is substituted by a linear equivalent or fictitious material, respectively. The resulting methodologies have been applied to additively manufactured ABS U-notched single-edge-notched bending (SENB) specimens combining five different notch radii (0, 0.25, 0.5, 1 and 2 mm) and three different raster orientations (0/90, 45/−45 and 30/−60). The results obtained demonstrate that both the ASED-EMC and the ASED-FMC combined criteria provide more accurate predictions than those obtained directly through the ASED criterion, with the ASED-EMC criterion generally providing safe more accurate predictions, with an average deviation from the experimental fracture loads between +1.0% (predicted loads higher than experimental loads) and −7.6% (predicted loads lower than experimental loads).

## 1. Introduction

Additive manufacturing (AM) is a growing technology with the potential to change the way fabrication and production processes are developed nowadays. Its capacity to make complex parts or designs in a relatively simple way using a wide variety of materials (e.g., polymers, metals, ceramics etc.) makes it a powerful tool. This work is focused on fused deposition modelling (FDM), one of the most important AM alternatives within Material Extrusion (ME) technology, and its capacity to manufacture acrylonitrile butadiene styrene (ABS) parts. FDM consists in extruding a plasticised filament through a heated nozzle, which is deposited on a build platform layer by layer until the final product is made [1]. This allows a computer-aided design (CAD) model to be converted into a real component with high levels of precision and in a relatively fast process. However, FDM has been mostly used for the prototyping of components, and not for final components with structural responsibilities. The main reason for this is that 3D (and FDM) printed components generally present lower mechanical properties than those achieved by traditional methods (e.g., injection, extrusion, casting, etc.). In this regard, the scientific community has been making great efforts to gain an improved understanding of the impact of FDM parameters on the mechanical properties (especially tensile properties) of the final material. For example, it has been widely observed how the building directions and raster orientations affect the final tensile properties (e.g., [1,2,3,4]). In terms of fracture properties, layer and build orientations and infill levels were found to be the major factors controlling the fracture toughness of AM parts fabricated through FDM processes (e.g., [4,5,6,7,8,9]).

As mentioned above, one of the advantages of AM is its capacity to manufacture complex parts. These parts may contain a wide variety of stress risers, such as defects generated during the manufacturing process (such as warping, poor surface finish or porosity [10,11,12,13,14,15,16,17,18]), defects caused by operational damage, or structural details included in the original design (e.g., notches, holes, corners, cut-outs, etc.). These types of stress risers may be analysed as cracks when following traditional fracture mechanics criteria. However, it has been widely demonstrated how non-sharp stress risers (here referred to as notches) make the corresponding material behave as if it had an apparent fracture toughness which is generally higher than the material fracture toughness obtained from laboratory specimens containing sharp defects (i.e., cracks). Thus, treating notches as if they were cracks tends to provide over-conservative analyses. In order to avoid this over-conservatism, different methodologies may be applied to take into consideration the notch effect (e.g., [19,20,21,22,23,24,25,26,27,28,29]): one example is the global criterion [19,20,21], which is based on linear-elastic notch fracture mechanics, and establishes that a fracture occurs when the notch stress intensity factor reaches a critical value. Other examples are the local criteria, based on the analysis of the stress, strain or energy fields at the defect tip, of which the Theory of Critical Distances (TDC) [22] or the Average Strain Energy Density (ASED) [23] can be highlighted. Finally, the progressive damage models [24,25,26] consider the material damage during the entire loading process and the consequent changes in the stress distribution.

The ASED criterion has been validated in numerous materials with brittle or quasi-brittle behaviour and different loading conditions (e.g., [27,28,29]). In a recent publication, Seibert et al. [30] successfully applied the ASED criterion in additive manufactured polylactic acid (PLA) material by using an alternative approach to determine the control volume used in this criterion (see Section 2). Alternatively, in the presence of non-linear behaviour, Torabi proposed the Equivalent Material Concept (EMC) [31], with the idea of transforming a non-linear material (in terms of tensile behaviour) into an equivalent linear-elastic material and allowing the use of the corresponding (generally simpler) elastic assessment tools (e.g., TDC [32], ASED [32,33], Maximum Tangential Stress [34], etc.). However, the EMC was not accurate enough for materials that present non-linear behaviour in both the tensile and the fracture behaviours, for which the same author developed the Fictitious Material Concept (FMC) [35], with a similar purpose to that of the EMC. Since the moment when these methodologies (ASED, EMC and FMC) were conceptualized, their validation has been extended not only to metals but also to polymeric and composite materials [33,34,36,37,38,39,40].

With all this, this paper provides an approach to the analysis of additively manufactured (FDM) ABS material containing U-notches by combining the ASED criterion with the EMC and the FMC corrections. To the author´s knowledge this is the first attempt to apply this kind of approach on AM polymer-matrix materials. Thus, Section 2 provides the theoretical framework of the research, including a description of the ASED criterion, the EMC and the FMC. Section 3 presents the materials and methods used for the prediction of the critical loads. Section 4 gathers the experimental results and provides the predictions of the critical loads obtained through the proposed approach, together with the corresponding discussion. Finally, Section 5 presents the main conclusions.

## 2. Theoretical Background

### 2.1. Average Strain Density Criterion

The Average Strain Energy Density (ASED) criterion, whose final bases were developed by Lazzarin and Berto [23,41,42,43], establishes that brittle failure occurs when the mean value of the strain energy density ($\bar{W}$) over a control volume (or an area in two-dimensional cases) is equal to the critical energy (W_c_) (1) [41]:

(1) $$\bar{W}=W_{c}$$

W_c_ is a material property which, in the case of a lineal-elastic material, may be directly derived as a function of the ultimate tensile strength (σ_u_) and Young´s modulus (E) (2):

(2) $$W_{c}=\frac{\sigma_{u}^{2}}{2E}$$

**Figure 1:**
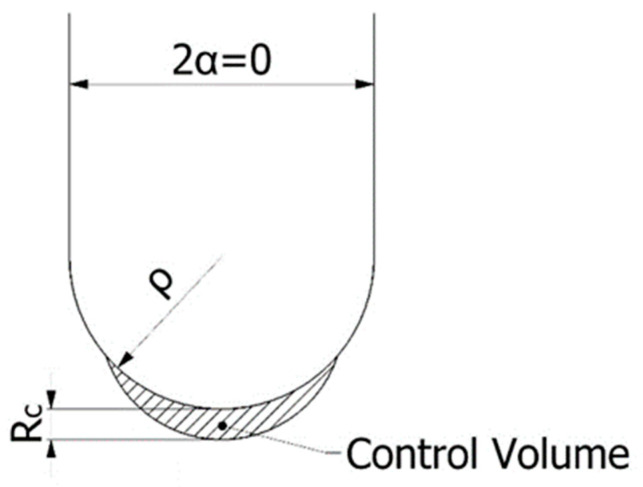
Control volume (area) for U-notch (2α = 0) under mode I loading.

In a plane case, the control volume becomes a circular sector with a critical radius R_c_. This parameter varies with the notch-opening angle (α). However, for the U-notches analysed in this work, 2α = 0 (see Figure 1), and Yosibash et al. [44] have developed very useful expressions for R_c_. When the fracture toughness reaches the limit imposed by Equation (3) [22], plane strain conditions dominate. Under this situation, R_c_ can be expressed as Equation (4) [44].

(3) $$K_{c}<\sigma_{y}{\left(\frac{B}{2.5}\right)}^{0.5}$$

(4) $$R_{c}=\frac{\left(1+v\right)\left(5-8v\right)}{4\pi }{\left(\frac{K_{c}}{\sigma_{u}}\right)}^{2}$$

σ_y_ being the yield strength, B the specimen thickness, K_c_ the fracture toughness, σ_u_ the ultimate tensile strength and ν the Poisson´s ratio. On the other hand, plane stress conditions are reached when the fracture toughness exceeds the limit defined by Equation (5) [22], with R_c_ following Equation (6) [44]. In those situations where K_c_ is found between the two limits, an interpolation between Equations (4) and (6) is required to determine R_c_.

(5) $$K_{c}>\sigma_{y}{\left(\pi B\right)}^{0.5}$$

(6) $$R_{c}=\frac{\left(5-3v\right)}{4\pi }{\left(\frac{K_{c}}{\sigma_{u}}\right)}^{2}$$

Finally, the mean value of the strain energy density (enclosed within the R_c_), may be directly obtained by the following analytical expression (7) [23]:

(7) $$\bar{W}=F\left(2\alpha \right)H\left(2\alpha ,\frac{R_{c}}{\rho }\right)\frac{\sigma_{\max}^{2}}{E}$$

F being a function that depends on the notch opening angle (2α), whose values are reported in [23]. When the opening angle is zero (U-notches), F takes a value of 0.785. The function H depends on the notch opening angle and the ratio of critical radius to the notch radius, with some values being gathered in Table 1 [23]. Finally, σ_max_ corresponds to the maximum principal stress at the notch tip. Thus, it is worth noticing that the ASED criterion may be supported by numerical methods, providing a powerful engineering tool to predict failure loads on complex parts, as long as the material being analysed exhibits brittle (linear-elastic) behaviour.

### 2.2. The Equivalent and Fictitious Material Concepts

As briefly introduced above, the main assumptions of both the EMC and FMC are that they equate a real ductile material to a virtual brittle one, to subsequently apply well-known linear-elastic fracture criteria (e.g., ASED, TDC, etc.).

**Figure 2:**
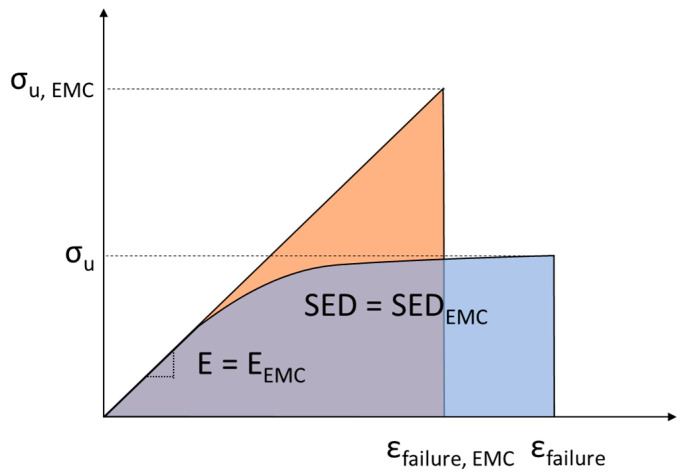
Tensile stress-strain curves for the real material (ductile) and the EMC material (linear-elastic).

The EMC was proposed for materials with limited ductility in the tensile behaviour [31] and linear-elastic behaviour at the onset of fracture. Thus, it is necessary only to replace the (non-linear) tensile curve by a perfectly linear (virtual) curve, with the corresponding equivalent tensile strength (σ_u,EMC_), and keeping the same Young´s modulus of the real material (see Figure 2).

On the other hand, the FMC aims to correct non-linear materials in both tensile and fracture conditions. In order to do this, it is necessary to determine two essential parameters, a fictitious tensile strength (σ_u,FMC_) and a fictitious fracture toughness (K_c,FMC_) [35].

**Figure 3:**
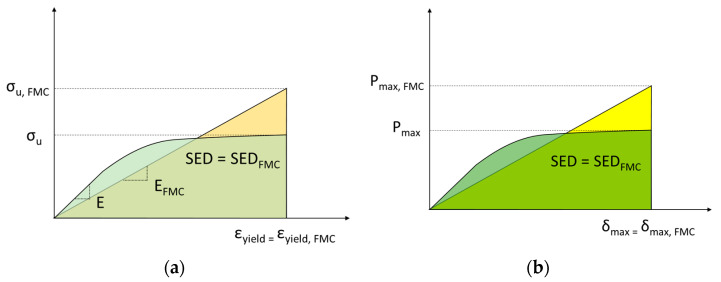
Real material and FMC material; (**a**) Tensile stress-strain curves; (**b**) Load-displacement curves of pre-cracked samples in fracture tests.

In order to calculate the tensile strength of the (virtual) brittle material, it is assumed that the two materials absorb the same amount of SED at failure. In addition, the FMC stipulates that the ductile materials and the virtual material have a different Young’s modulus, but the strain under the maximum stress is the same. This important assumption means that the tensile strength of the fictitious material may be higher than the ultimate tensile strength of the real ductile material (see FMC scheme in Figure 3a). Here it is worth mentioning that a significant difference between the EMC and the FMC lies in the fact that the EMC assumes the same value of Young´s modulus for both the real ductile and the fictitious brittle material, but the strain at failure has to be different, as is shown in Figure 2.

The fracture toughness for the fictitious material may be easily calculated through the real load-displacement curves of the real material pre-cracked specimens. According to FMC, it is assumed (again) that the strain energy density (SED) required to achieve the onset of fracture in the real ductile material is equal to that developed in the fictitious brittle material. Moreover, the displacement values at fracture in both the real ductile material and the fictitious brittle material are the same, as shown in Figure 3b. With all this, the value of the fictitious maximum load (P_max,FMC_) at fracture may be easily calculated, from which the fracture toughness of the fictitious material (K_c,FMC)_ may be directly obtained from well-known linear-elastic fracture mechanics formulations [35].

In either of the two approaches (EMC or FMC), the tensile behaviour of a ductile material can be expressed by Hollomon´s Equation (8) [31]:

(8) $$\sigma ={K\varepsilon }_{p}^{n}$$

σ, K, n and $\varepsilon_{p}^{n}$ being the true stress, the strength coefficient, the strain-hardening exponent and the true plastic strain, respectively.

In a ductile material, failure initiates when the maximum load is reached. At that moment the corresponding SED is calculated by integrating the stress-strain curve from the origin until the maximum load, on which $\varepsilon_{p}^{n}$ is denoted as ε_u,True_. The total SED until the maximum stress (area below the tensile curve) may then be expressed as (9) [31]:

(9) $${\left(\mathrm{SED}\right)}_{\mathrm{total}}=\frac{\sigma_{y}^{2}}{2E}+\frac{K}{n+1}\left(\varepsilon_{u,\mathrm{True}}^{n+1}-{\left(0.002\right)}^{n+1}\right)$$

Now, the total SED value until the maximum stress for the virtual brittle material is defined by Equation (10) [31] for the EMC, and Equation (11) [35] in the case of the FMC (again, in both cases, it is calculated as the area below the resulting linear-elastic tensile curves).

(10) $${\left(\mathrm{SED}\right)}_{\mathrm{EMC}}=\frac{\sigma_{u,\mathrm{EMC}}{}^{2}}{2E}$$

(11) $${\left(\mathrm{SED}\right)}_{\mathrm{FMC}}=\frac{1}{2}\varepsilon_{u}\sigma_{u,\mathrm{FMC}}$$

Therefore, considering that the SED value is equal for the real and the virtual materials, Equation (9) may be combined with Equations (10) and (11), deriving the values of σ_u,EMC_ and σ_u,FMC_ from Equations (12) [31] and (13) [35], respectively.

(12) $$\sigma_{u,\mathrm{EMC}}=\sqrt{\sigma_{y}^{2}+\frac{2\mathrm{EK}}{n+1}\left({\left(\varepsilon_{u,\mathrm{True}}\right)}^{\left(n+1\right)/n}-{\left(0.002\right)}^{n+1}\right)}$$

(13) $$\sigma_{u,\mathrm{FMC}}=\frac{\sigma_{y}^{2}}{\varepsilon_{u,\mathrm{True}}E}+\frac{2K}{\varepsilon_{u,\mathrm{True}}\left(n+1\right)}\left(\varepsilon_{u}{}^{n+1}-{\left(0.002\right)}^{n+1}\right)$$

To sum up, both the EMC and the FMC substitute the real ductile material by a virtual linear-elastic material. The EMC only modifies the tensile strength (Equation (12)) considering that the real and the virtual materials develop the same SED at failure, maintaining the same E [31]; the FMC modifies both the tensile strength and the material toughness. For the tensile strength, the strain at failure of the real material and the virtual material are the same, and assuming that the SED at failure is also equal, the tensile strength is derived from Equation (13). Concerning the fracture toughness, the FMC also considers the same SED and displacement at failure for the real and the virtual materials, and assuming linear-elastic behaviour of the virtual material, the fracture toughness is easily derived [35].

## 3. Materials and Methods

In this work, an AM ABS material was analysed. With this aim, a series of tensile and fracture specimens were completed. All specimens were printed using a Prusa i3 printer with the following printing parameters: layer height: 0.3 mm; line width: 0.4 mm; infill degree: 100%; printing temperature: 230 °C; bed temperature: 95 °C and printing rate: 40 mm/s. In addition, three different raster orientations were studied: 0/90, 45/−45 and 30/−60.

**Figure 4:**
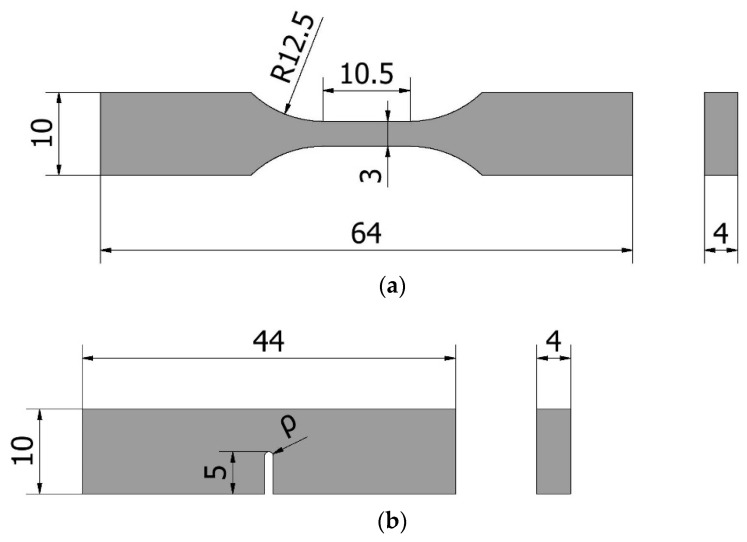
The geometry of the ABS AM specimens: (**a**) tensile specimens; (**b**) fracture specimens, with ρ varying from 0 mm up to 2 mm. Dimensions in mm.

The tensile behaviour was characterised by testing nine specimens, with their geometry being shown in Figure 4a. The tests were performed at room temperature (20 °C) and an approximate humidity of 57%, with a loading rate of 5 mm/min, following the guidelines established by ASTM D638 [45]. The applied load, as well as the elongation (measured by an axial extensometer with a 12.5 mm gauge length) were continuously recorded.

For fracture characterisation, 33 single-edge notch bending (SENB) specimens were tested (see Figure 4b). A total of five different notch radii were considered: 0 mm (crack-like defect), 0.25 mm, 0.5 mm, 1 mm and 2 mm. The notches were all machined, except for the crack-like defects, which were generated by sawing using a razor blade. Fracture tests were carried out at room temperature (20 °C) and about 57% humidity applying a loading rate of 10 mm/min according to the standard ASTM D6068 [46]. Fracture toughness values were initially calculated following linear-elastic assumptions and the standard ASTM D5045 [47]. However, given that some of the validity criteria of this standard (e.g., P_max_/P_Q_ < 1.1, see Section 9.1.1 in [47]) were not fulfilled in many cases, fracture toughness values were also calculated using ASTM D6068, assuming that there was no stable crack propagation before the final fracture. Further details about the experimental program and the materials may be found in [5].

Once the ABS material was characterised in terms of tensile and fracture properties, the parameters of the EMC (σ_u,EMC_) and the FMC (σ_u,FMC_, K_c,FMC_) were calibrated following the procedure described in Section 2.2 [31,35]. Once the corresponding linear-elastic (virtual) material was defined, the final step was to apply the linear-elastic ASED criterion to predict the failure load of each SENB specimen. As mentioned above, the average control volume depends on the stress field at the defect tip. Thus, according to the ASED failure criteria and following Equations (1), (2) and (7), the maximum stress at the notch tip may be easily derived. Then, considering that σ_max_ (in mode I) is reached in the notch root (r = 0), and applying the U-notch stress distribution proposed by Creager–Paris [48], the stress intensity factor (K_I_) may be straightforwardly derived from Equation (14). Finally, using the well-known analytical solution of the K_I_ for SENB specimens (Equation (15)) [49], the values of the predicted failure loads (P_critical_) were obtained.

(14) $$\sigma \left(r=0\right)=\sigma_{\max}=\frac{2K_{I}}{\sqrt{\pi \rho }}$$

(15) $$K_{I}=\left(\frac{P_{\mathrm{critical}}}{{\mathrm{BW}}^{0.5}}\right)\cdot 6\cdot {\left(\frac{a}{W}\right)}^{0.5}\left(\frac{1.99-\left(\frac{a}{W}\right)\cdot \left(1-\frac{a}{W}\right)\cdot \left(2.15-3.93\left(\frac{a}{W}\right)+2.7{\left(\frac{a}{W}\right)}^{2}\right)}{\left(1+2\frac{a}{W}\right)\cdot {\left(1-\frac{a}{W}\right)}^{1.5}}\right)$$

where a, B, and W denote the crack length, the specimen thickness, and the specimen width, respectively.

## 4. Results and Discussion

**Figure 5:**
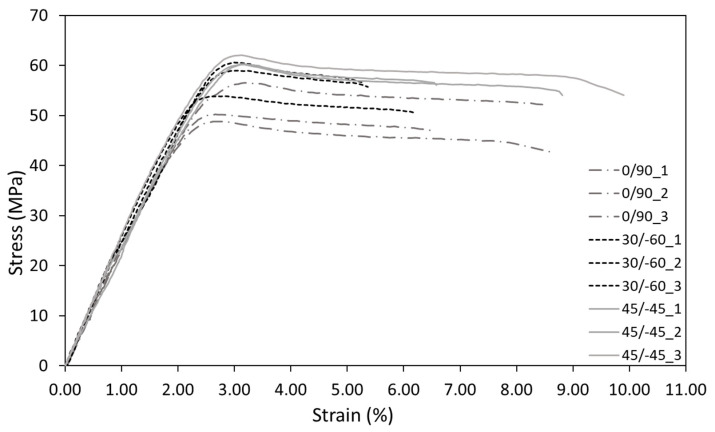
The stress-strain curves of the AM ABS material for the three different raster orientations.

The results of the tensile tests are gathered in Table 2, while the stress-strain curves per raster orientation are presented in Figure 5. The 45/−45 configuration presents greater values of the Young´s modulus and elongation at yield, with the 0/90 orientation providing the lower values. In all three orientations, two distinct regions can be observed. Initially, there is an elastic response up to approximately a level of strain between 2% and 3%, with clear non-linear behaviour before the maximum stress, which following ASTM D638 [45] is considered the material strength at yield (σ_sy_). This value is followed by material softening, with the strains being homogeneously distributed along the gauge length and without any indication of necking. Finally, the material breaks at the tensile stress at break [45]. Here it should be noted that the second region (from the material strength at yield until failure) does not contribute to the load-bearing capacity and, thus, only the first area of the curve should be considered for load-bearing capacity analyses using the ASED criterion. Furthermore, following ASTM D638, the maximum stress (σ_sy_) coincides with the yield point and, consequently, with the yield strength (σ_y_ = σ_sy_). σ_sy_ and ε_yield_ are then used as σ_u_ and ε_u_ (respectively) to calibrate the EMC and the FMC approaches.

**Figure 6:**
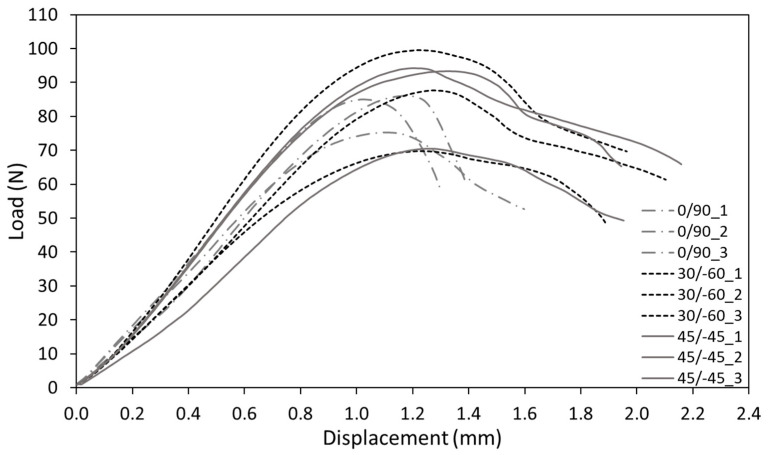
Load-displacement curves of the AM ABS for the three different raster orientations.

Regarding the fracture behaviour, the load-displacement curves obtained in cracked conditions are shown in Figure 6, which are then employed to calculate the fracture resistance (K_c_) for the conditions here analysed. Details about the fracture micromechanisms may be found in [5]. The results of K_c_ for the different orientations are also gathered in Table 2. These values were evaluated by following the ASTM D5045 standard [47], although in certain cases the ratio P_max_ /P_Q_ was slightly higher than 1.1 (see Section 9.11 in [47] for details). The average ratios of P_max_/P_Q_ for the three different raster orientations were 1.08, 1.17 and 1.18 for 0/90, 30/−60 and 45/−45, respectively. In any case, by applying ASTM D5045 the results are always on the safe side.

The results obtained here both in tensile and fracture conditions are similar, but slightly higher, than those found in the literature (e.g., [4,50]).

Finally, it is worth mentioning that the analysed materials are anisotropic. Therefore, the characterization performed here allows performing the analyses on this particular loading and crack propagation directions.

### ASED-EMC and ASED-FMC Fracture Load Predictions

Table 3 gathers the calibrated parameters of the EMC and the FMC for each raster orientation.

As mentioned above, the application of the ASED criterion depends on a variety of material properties, such as fracture toughness, ultimate tensile strength, Young’s modulus, and Poisson’s ratio. While the first three parameters are directly derived from the approaches being applied (EMC, FMC), the Poisson’s ratio is considered to be equal in the real and the virtual materials. However, it was not determined experimentally in the original test program. Therefore, according to the literature, this parameter was considered to be 0.35 [1,50,51]. In any case, a sensitivity analysis was performed to examine how Poisson’s ratio affects the final prediction, revealing that the results are not particularly sensitive to the specific value used in this parameter (for example, the greatest variation in the critical load predictions when considering a Poisson’s ratio of 0.4 is about 2%).

**Figure 7:**
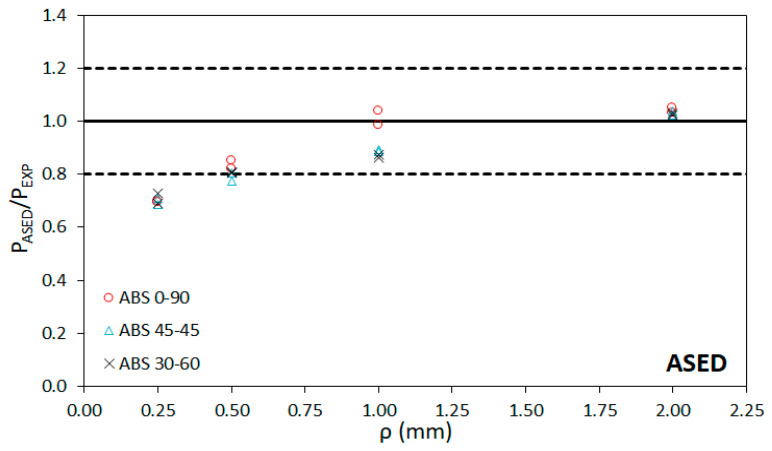
Comparison between fracture load predictions using ASED and experimental fracture loads.

**Figure 8:**
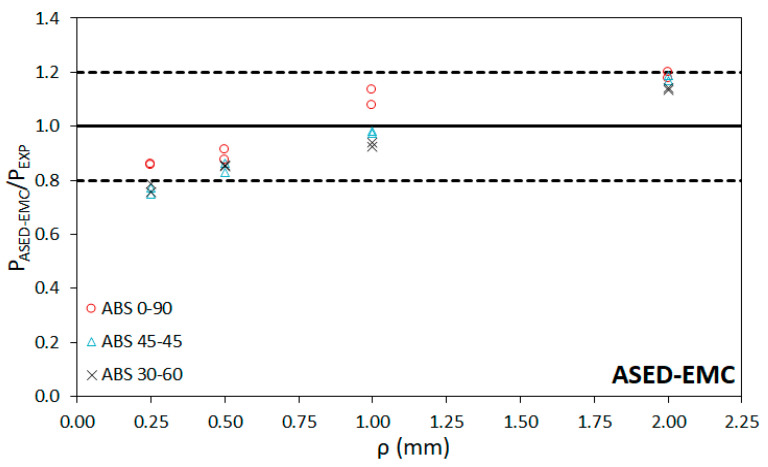
Comparison between fracture load predictions using ASED-EMC and experimental fracture loads.

**Figure 9:**
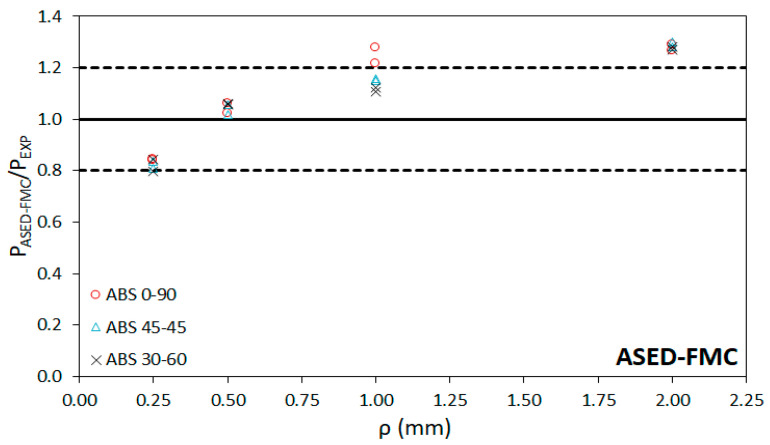
Comparison between fracture load predictions using ASED-FMC and experimental fracture loads.

Table 4 presents the critical load predictions obtained after applying the ASED-EMC and ASED-FMC approaches, together with their corresponding deviations from the physical value. These predictions are obtained by applying Equations (1) to (7) and considering the material properties of the virtual (equivalent of fictitious) materials gathered in Table 3. Additionally, Table 4 includes the fracture load predictions by directly using the ASED criterion, without any correction (i.e., using the properties of the non-linear real materials). It can be observed that the average deviation ranges between −14.9% and−10.5% for the ASED model, between 1.0% and −7.6% for the ASED-EMC model, and between 6.7% and 10.2% in the case of the ASED-FMC model. Here, negative deviations (or errors) mean that the predictions of the critical loads underestimate the real critical loads, whereas positive deviations imply predictions that are higher than the real loads (non-conservative). The lowest accuracy of the predictions is usually found in the specimens with 0.25 mm of notch tip radius (negative errors above −30%) and with 2.0 mm of notch tip radius (up to +30% of error). The latter cases may be mainly caused by the loss of accuracy of the Creager–Paris expression to determine the stress distribution (Creager–Paris solutions require slender notches in which the notch length is significantly larger than the notch radius). Figure 7, Figure 8 and Figure 9 compare the predicted results (P_ASED_, P_ASED-EMC_ or P_ASED-FMC_) with the experimental loads (P_EXP_) along with the ±20% lines, which represents a commonly accepted deviation in fracture research [25,28,30]. In general, it seems that the ASED-EMC model provides more accurate conservative predictions than the ASED-FMC approach, although this model seems to be more accurate for the lower notch radii (0.25 mm). In both cases, predictions are conservative for lower notch radii (0.25 mm and 0.5 mm) and become non-conservative for the larger radii (1 mm and 2 mm).

As described above, the biggest discrepancy between the EMC and FMC is the necessity to calibrate the fracture resistance in the second model. Furthermore, in the AM ABS material analysed here, there is the peculiarity that the value of the ultimate strength of the virtual material obtained by both approaches is almost the same (see Table 3) while the literature shows that the FMC generates lower strengths than the EMC [38]. In any case, the real material is very close to the validity range limits of linear-elastic fracture mechanics characterisation tools, so the need to provide corrections to the fracture properties (through the FMC) is not completely justified. In other words, the ASED-EMC methodology appears to be the most optimal method due to the limited non-linear behaviour developed by the AM ABS material.

## 5. Conclusions

This paper contributes to the development of suitable failure prediction models to analyse and estimate the critical fracture loads of cracked and notched additively manufactured ABS components. The ABS material analysed here does not develop fully linear-elastic behaviour, so the application of linear-elastic tools requires certain previous corrections. In this sense, a couple of approaches proposed by Torabi, the equivalent material concept (EMC) and the fictitious material concept (FMC), were combined with a well-known linear-elastic criterion, the average strain energy density (ASED) method. The resulting methodologies, ASED-EMC and ASED-FMC, were applied to U-notched AM ABS specimens with three different raster orientations (0/90, 45/−45, 30/−60). The main conclusions can be summarised as follows:

- The strict application of the ASED criterion provides the most conservative results, since it was formulated for brittle materials with linear elastic behavior.
- Both the ASED-EMC and the ASED-FMC criteria improves the accuracy of the predictions provided by the ASED approach.
- The ASED-EMC criterion has been the best approach to predict the failure loads in AM ABS material containing U-notches, generally combining accuracy and safety.
- Thus, a powerful engineering tool that may avoid time-consuming elastoplastic analyses has been validated.

## Figures and Tables

**Table 1:** Some values of the H function for U-shaped notches [23].

Rc/ρ	H		
	ν = 0.3	ν = 0.35	ν = 0.4
0.01	0.5638	0.5432	0.5194
0.05	0.5086	0.4884	0.4652
0.1	0.4518	0.4322	0.4099
0.3	0.3069	0.2902	0.2713
0.5	0.2276	0.2135	0.1976
1	0.1314	0.1217	0.1110

**Table 2:** ABS AM mechanical properties; E, Young´s modulus; σ_sy_, tensile strength at yield; ε_yield_, strain at yield; SED, strain energy density; K_c_, fracture resistance.

	0/90		30/−60		45/−45	
Material Property	Mean	Std. Dev.	Mean	Std. Dev.	Mean	Std. Dev.
E (MPa)	2241	169.34	2329	45.45	2388	181.67
σ_sy_ (MPa)	51.77	4.08	59.37	1.10	60.87	1.07
ε_yield_ (%)	2.90	0.30	2.92	0.22	3.14	0.02
SED (MPa)	0.91	0.15	1.01	0.06	1.13	0.04
K_c_ (MPa·m^0.5^)	2.05	0.16	1.99	0.35	2.03	0.16

**Table 3:** Properties of the virtual (brittle) material according to EMC and FMC for the different raster orientations.

	0/90		30/−60		45/−45	
Material Property	Mean	Std. Dev.	Mean	Std. Dev.	Mean	Std. Dev.
σ_u,EMC_ (MPa)	62.67	4.36	68.54	1.26	71.62	2.88
σ_u,FMC_ (MPa)	63.64	5.15	68.64	2.18	73.34	1.34
K_c,FMC_ (MPa·m^0.5^)	2.55	0.15	2.75	0.48	2.80	0.47
E_FMC_	2176	59.80	2323	53.63	2276	100.93

**Table 4:** Experimental critical loads (P_exp_) of the fracture specimens along with the predictions obtained using ASED, ASED-EMC and ASED-FMC approaches.

Raster Orientation	N° Test	ρ (mm)	P_EXP_ (N)	P_ASED_ (N)	P_ASED-EMC_ (N)	P_ASED-FMC_ (N)	Deviation ASED (%)	Deviation ASED-EMC (%)	Deviation ASED-FMC (%)
0/90	1	0.25	89.9	62.3	76.8	75.5	−30.8	−14.6	−16.1
	2	0.25	89.5	62.3	76.8	75.5	−30.4	−14.2	−15.7
	3	0.50	98.2	80.3	85.8	100.4	−18.2	−12.6	2.3
	4	0.50	93.6	79.6	85.3	99.3	−15.0	−8.9	6.0
	5	1.00	85.9	89.2	97.4	109.9	3.7	13.4	28.0
	6	1.00	90.5	89.2	97.5	109.9	−1.5	7.7	21.5
	7	2.00	101.3	106.4	121.3	130.5	5.0	19.7	28.8
	8	2.00	102.9	106.3	121.2	130.4	3.2	17.7	26.6
Average Error							−10.5	1.0	10.2
30/−60	1	0.25	103.4	71.4	78.2	82.9	−30.9	−24.4	−20.2
	2	0.25	97.8	71.4	77.0	82.9	−27.0	−21.3	−15.6
	3	0.50	100.4	81.4	86.1	106.4	−19.0	−14.3	6.0
	4	0.50	101.1	81.5	86.2	106.8	−19.4	−14.8	5.6
	5	1.00	107.7	94.0	101.0	121.1	−12.7	−6.2	12.2
	6	1.00	110.2	94.8	101.7	122.2	−14.0	−7.7	10.7
	7	2.00	111.9	114.6	127.1	142.4	2.4	13.5	26.9
	8	2.00	111.6	115.1	127.5	143.3	3.2	14.2	28.1
Average Error							−14.7	−7.6	6.7
45/−45	1	0.25	103.2	73.2	79.7	86.2	−29.05	−22.8	−16.4
	2	0.25	106.5	73.2	79.8	86.2	−31.28	−25.1	−19.0
	3	0.50	108.2	83.7	89.9	110.3	−22.66	−16.9	1.9
	4	0.50	103.3	83.2	89.3	109.2	−19.48	−13.5	5.7
	5	1.00	108.8	96.2	105.8	124.9	−11.56	−2.8	14.8
	6	1.00	107.6	95.9	105.5	124.5	−10.83	−1.9	15.7
	7	2.00	112.9	117.2	134.1	147.0	3.81	18.8	30.2
	8	2.00	114.8	117.4	134.2	147.3	2.26	17.0	28.3
Average Error							−14.9	−5.9	7.7

## Data Availability

The data presented in this study are available on request from the corresponding author.

## Nomenclature

ABS	Acrylonitrile Butadiene Styrene
PLA	Polylactic Acid
AM	Additive Manufacturing
ME	Material Extrusion
FDM	Fused Deposition Modeling
CAD	Computer-Aided Design
TDC	Theory of Critical Distances
ASED	Average Strain Energy Density
SED	Strain Energy Density
EMC	Equivalent Material Concept
FMC	Fictitious Material Concept
SENB	Single Edge Notched Bending (specimen)
$\bar{W}$	mean value of the strain energy density
W_c_	critical strain energy density
R_c_	critical radius
σ_u_	ultimate tensile strength
σ_y_	yield strength
σ_sy_	tensile strength at yield
ε_yield_	strain at yield
E	Young’s modulus
ν	Poisson´s ratio
K_c_	material fracture toughness in stress intensity factor units
B	specimen thickness
K_I_	applied stress intensity factor
σ_max_	maximum principal stress at the notch tip
σ_u,EMC_	equivalent tensile strength
σ_u,FMC_	fictitious tensile strength
K_c,FMC_	fictitious fracture toughness in stress intensity factor units
E_FMC_	fictitious Young´s modulus
P_max,FMC_	fictitious maximum load
P_critical_	predicted failure load
P_max_	maximum load
P_Q_	load provided by the intersection of the load-displacement curve and a line with a compliance 5% greater than the straight part of the load-displacement curve.
ρ	notch radius

## References

[1] Cantrell J.T., Rohde S., Damiani D., Gurnani R., DiSandro L., Anton J., Young A., Jerez A., Steinbach D., Kroese C. (2017). Experimental characterization of the mechanical properties of 3D-printed ABS and polycarbonate parts. Rapid Prototyp. J..

[2] Bamiduro O., Owolabi G., Haile M.A., Riddick J.C. (2019). The influence of load direction, microstructure, raster orientation on the quasi-static response of fused deposition modeling ABS. Rapid Prototyp. J..

[3] Ahn S., Montero M., Odell D., Roundy S., Wright P.K. (2002). Anisotropic material properties of fused deposition modeling ABS. Rapid Prototyp. J..

[4] Ng C.T., Susmel L. (2020). Notch static strength of additively manufactured acrylonitrile butadiene styrene (ABS). Addit. Manuf..

[5] Cicero S., Martínez-Mata V., Alonso-Estebanez A., Castanon-Jano L., Arroyo B. (2020). Analysis of Notch Effect in 3D-Printed ABS Fracture Specimens Containing U-Notches. Materials.

[6] Ameri B., Taheri-Behrooz F., Aliha M.R.M. (2020). Fracture loads prediction of the modified 3D-printed ABS specimens under mixed-mode I/II loading. Eng. Fract. Mech..

[7] Isaac J.P., Dondeti S., Tippur H.V. (2021). Fracture behavior of additively printed ABS: Effects of print architecture and loading rate. Int. J. Solids Struct..

[8] McLouth T.D., Severino J.V., Adams P.M., Patel D.N., Zaldivar R.J. (2017). The impact of print orientation and raster pattern on fracture toughness in additively manufactured ABS. Addit. Manuf..

[9] Hart K.R., Wetzel E.D. (2017). Fracture behavior of additively manufactured acrylonitrile butadiene styrene (ABS) materials. Eng. Fract. Mech..

[10] Abdulhameed O., Al-Ahmari A., Ameen W., Mian S.H. (2019). Additive manufacturing: Challenges, trends, and applications. Adv. Mech. Eng..

[11] Haidiezul A., Aiman A., Bakar B. (2018). Surface Finish Effects Using Coating Method on 3D Printing (FDM) Parts. IOP Conf. Ser. Mater. Sci. Eng..

[12] Tang Q., Yin S., Zhang Y., Wu J. (2018). A tool vector control for laser additive manufacturing in five-axis configuration. Int. J. Adv. Manuf. Technol..

[13] Kousiatza C., Karalekas D. (2016). In-situ monitoring of strain and temperature distributions during fused deposition modeling process. Mater. Des..

[14] Syrlybayev D., Zharylkassyn B., Seisekulova A., Akhmetov M., Perveen A., Talamona D. (2021). Optimisation of Strength Properties of FDM Printed Parts—A Critical Review. Polymers.

[15] Wong K.V., Hernandez A. (2012). A Review of Additive Manufacturing. ISRN Mech. Eng..

[16] Agron D.J.S., Nwakanma C.I., Lee J.-M., Kim D.-S. (2020). Smart Monitoring for SLA-type 3D Printer using Artificial Neural Network. KICS.

[17] Kadam V., Kumar S., Bongale A., Wazarkar S., Kamat P., Patil S. (2021). Enhancing Surface Fault Detection Using Machine Learning for 3D Printed Products. Appl. Syst. Innov..

[18] Ameri B., Taheri-Behrooz F., Aliha M.R.M. (2021). Evaluation of the geometrical discontinuity effect on mixed-mode I/II fracture load of FDM 3D-printed parts. Theor. Appl. Fract. Mech..

[19] Beaumont P.W.R. (1989). The failure of fibre composites: An overview. J. Strain Anal. Eng. Des..

[20] Head P. (1996). Advanced composite materials. Eng. Des..

[21] Bäcklund J., Aronsson C.G. (1986). Tensile Fracture of Laminates with Holes. J. Compos. Mater..

[22] Taylor D. (2007). The Theory of Critical Distances. Theory Crit. Distances.

[23] Berto F., Lazzarin P., Ayatollahi M.R. Recent developments in brittle and quasi-brittle failure assessment of graphite by means of SED approach. Proceedings of the 13th International Conference on Fracture.

[24] Nguyen B.N. (1997). Three-dimensional modeling of damage in laminated composites containing a central hole. J. Compos. Mater..

[25] Chang K.Y., Llu S., Chang F.K. (1991). Damage Tolerance of Laminated Composites Containing an Open Hole and Subjected to Tensile Loadings. J. Compos. Mater..

[26] Lawcokc G., Lin Y., Mai Y. (1997). Progressive Damage and Residual Strength of a Carbon Fibre Reinforced Metal Laminate. J. Compos. Mater..

[27] Justo J., Castro J., Cicero S. (2018). Energy-based approach for fracture assessment of several rocks containing U-shaped notches through the application of the SED criterion. Int. J. Rock Mech. Min. Sci..

[28] Ibáñez-Gutiérrez F.T., Cicero S., Madrazo V., Berto F. (2018). Fracture Loads Prediction on Notched Short Glass Fibre Reinforced Polyamide 6 Using the Strain Energy Density. Phys. Mesomech..

[29] Cicero S., Berto F., Ibáñez-Gutiérrez F.T., Procopio I., Madrazo V. (2017). SED criterion estimations of fracture loads in structural steels operating at lower shelf temperatures and containing u-notches. Theor. Appl. Fract. Mech..

[30] Seibert P., Susmel L., Berto F., Kästner M., Razavi S.M.J. (2021). Applicability of strain energy density criterion for fracture prediction of notched PLA specimens produced via fused deposition modeling. Eng. Fract. Mech..

[31] Torabi A.R. (2012). Estimation of tensile load-bearing capacity of ductile metallic materials weakened by a V-notch: The equivalent material concept. Mater. Sci. Eng. A.

[32] Fuentes J.D., Cicero S., Berto F., Torabi A.R., Madrazo V., Azizi P. (2018). Estimation of fracture loads in AL7075-T651 notched specimens using the equivalent material concept combined with the strain energy density criterion and with the theory of critical distances. Metals.

[33] Cicero S., Fuentes J.D., Torabi A.R. (2020). Using the equivalent material concept and the average strain energy density to analyse the fracture behaviour of structural materials. Appl. Sci..

[34] Torabi A.R., Hamidi K., Rahimi A.S., Cicero S. (2021). Notch fracture in polymeric specimens under compressive stresses: The role of the equivalent material concept in estimating the critical stress of polymers. Appl. Sci..

[35] Torabi A.R., Kamyab M. (2019). The fictitious material concept. Eng. Fract. Mech..

[36] Torabi A.R., Rahimi A.S., Ayatollahi M.R. (2018). Fracture study of a ductile polymer-based nanocomposite weakened by blunt V-notches under mode I loading: Application of the Equivalent Material Concept. Theor. Appl. Fract. Mech..

[37] Cicero S., Torabi A.R., Majidi H.R., Gómez F.J. (2020). On the use of the combined FMC-ASED criterion for fracture prediction of notched specimens with nonlinear behavior. Procedia Struct. Integr..

[38] Torabi A.R., Majidi H.R., Cicero S., Ibáñez-Gutiérrez F.T., Fuentes J.D. (2019). Experimental verification of the Fictitious Material Concept for tensile fracture in short glass fibre reinforced polyamide 6 notched specimens with variable moisture. Eng. Fract. Mech..

[39] Cicero S., Torabi A.R., Madrazo V., Azizi P. (2018). Prediction of fracture loads in PMMA U-notched specimens using the equivalent material concept and the theory of critical distances combined criterion. Fatigue Fract. Eng. Mater. Struct..

[40] Albinmousa J., Alsadah J., Hawwa M.A., Al-Qahtani H.M. (2021). Estimation of mode I fracture of u-notched polycarbonate specimens using the equivalent material concept and strain energy density. Appl. Sci..

[41] Lazzarin P., Zambardi R. (2001). A finite-volume-energy based approach to predict the static and fatigue behavior of components with sharp V-shaped notches. Int. J. Fract..

[42] Berto F., Lazzarin P. (2009). A review of the volume-based strain energy density approach applied to V-notches and welded structures. Theor. Appl. Fract. Mech..

[43] Lazzarin P., Berto F. (2005). Some expressions for the strain energy in a finite volume surrounding the root of blunt V-notches. Int. J. Fract..

[44] Yosibash Z. (2012). Failure Criteria for Brittle Elastic Materials. Interdiscip. Appl. Math..

[45] (2014). Standard Test Method for Tensile Properties of Plastics.

[46] (2010). Standard Test Method for Determining J-R Curves of Plastic Materials.

[47] (2014). Standard Test Methods for Plane-Strain Fracture Toughness and Strain Energy Release Rate of Plastic Materials.

[48] Creager M., Paris P.C. (1967). Elastic field equations for blunt cracks with reference to stress corrosion cracking. Int. J. Fract. Mech..

[49] Anderson T.L. (2005). Fracture Mechanics: Fundamentals and Applications.

[50] Samykano M., Selvamani S.K., Kadirgama K., Ngui W.K., Kanagaraj G., Sudhakar K. (2019). Mechanical property of FDM printed ABS: Influence of printing parameters. Int J. Adv. Manuf. Technol..

[51] Dwiyati S.T., Kholil A., Riyadi R., Putra S.E. (2019). Influence of layer thickness and 3D printing direction on tensile properties of ABS material. J. Phys. Conf. Ser..

